# Sub-Tissue Localization of Phytochemicals in *Cinnamomum camphora* (L.) J. Presl. Growing in Northern Italy

**DOI:** 10.3390/plants10051008

**Published:** 2021-05-19

**Authors:** Martina Bottoni, Fabrizia Milani, Marta Mozzo, Daniele Armando Radice Kolloffel, Alessio Papini, Filippo Fratini, Filippo Maggi, Laura Santagostini

**Affiliations:** 1 Department of Pharmaceutical Sciences, University of Milano, Via Mangiagalli 25, 20133 Milano, Italy; martina.bottoni@unimi.it (M.B.); fabrizia.milani@unimi.it (F.M.); marta.mozzo@studenti.unimi.it (M.M.); 2 Ghirardi Botanic Garden, Department of Pharmaceutical Sciences, University of Milano, Via Religione 25, 25088 Toscolano Maderno, Brescia, Italy; 3 Department of Chemistry, University of Milano, Via Golgi 19, 20133 Milano, Italy; daniele.radicekolloffel@studenti.unimi.it; 4 Department of Biology, University of Florence, Via Micheli 3, 50121 Firenze, Italy; alessio.papini@unifi.it; 5 Department of Veterinary Sciences, University of Pisa, Viale delle Piagge 2, 56124 Pisa, Italy; filippo.fratini@unipi.it; 6 School of Pharmacy, University of Camerino, Via Sant’Agostino 1, 62032 Camerino, Italy; filippo.maggi@unicam.it

**Keywords:** camphor tree, Lauraceae, oil cells, essential oils, antibacterial activity

## Abstract

In the present paper, we focused our attention on *Cinnamomum camphora* (L.) J. Presl. (Lauraceae), studied at three levels: (i) micromorphological, with the analysis of the secretory structures and a novel in-depth histochemical characterization of the secreted compounds; (ii) phytochemical, with the characterization of the essential oils from young stems, fruits, and leaves, subjected to different conservation procedures (fresh, dried, stored at −20 °C, stored at −80 °C) and collected in two different years; (iii) bioactive, consisting of a study of the potential antibacterial activity of the essential oils. The micromorphological investigation proved the presence of secretory cells characterized by a multi-layered wall in the young stems and leaves. They resulted in two different types: mucilage cells producing muco-polysaccharides and oil cells with an exclusive terpene production. The phytochemical investigations showed a predominance of monoterpenes over sesquiterpene derivatives; among them, the main components retrieved in all samples were 1,8-cineole followed by *α*-terpineol and sabinene. Conservation procedures seem to only influence the amounts of specific components, i.e., 1,8-cineole and *α*-terpineol, while analyses on each plant part revealed the presence of some peculiar secondary constituents for each of them. Finally, the evaluation of the antibacterial activity of the essential oil showed a promising activity against various microorganisms, as *Listeria monocytogenes*, *Staphylococcus aureus*, *Enterococcus faecalis* and *Pseudomonas aeruginosa*. In conclusion, we combined a micromorphological and phytochemical approach of the study on different plant parts of *C. camphora*, linking the occurrence of secretory cells to the production of essential oils. We compared, for the first time, the composition of essential oils derived from different plant matrices conserved with different procedures, allowing us to highlight a relation between the conservation technique and the main components of the profiles. Moreover, the preliminary antibacterial studies evidenced the potential activity of the essential oils against various microorganisms potentially dangerous for plants and humans.

## 1. Introduction

*Cinnamomum camphora* (L.) J. Presl (Lauraceae), also known as a camphor tree, is native to tropical Asia, Malaysia and Taiwan, where it is preferably found at altitudes between 1000 and 1800 m asl. In the 17th century, it was introduced in Europe, where it is widely cultivated in gardens [1]. It is a very long-lived species that is able to adapt to different types of soil and climatic conditions [2]. It displays a solid stem, grey-brownish in color, with deep longitudinal furrows. The leaves are alternate, ovate-elliptic, oblong to lanceolate. Flowers, gathered in axillary inflorescences, are small, white or pale yellow; the fruits are globose drupes, black-purple in color at maturity [2].

In Chinese, Japanese and Indian Traditional Medicines, different parts of camphor tree were used for the preparation of essential oils and extracts employed for anti-inflammatory [3], antibacterial [4], antifungal [5,6], and antispasmodic purposes, for the treatment of febrile and convulsive states, in cases of toothache [7], and circulatory and respiratory apparatus diseases [8,9,10,11]. In addition, essential oils showed in vitro cytotoxic effects [6], central nervous system (CNS) stimulation properties [12], and mild analgesic action [13]. In cosmetics, essentials oils are used for manufacturing perfumes, creams, and balsamic ointments [3,11].

Despite the extensive scientific production related to the biological activity of *C. camphora*, a few contributions focused on morphological topics [1,14].

In the phytochemical field, the literature offers numerous contributions related to the analysis of the essential oil compositions of plants mainly grown in the native ranges [3,5,7,8,15,16,17]. As a whole, the profiles obtained by the different authors shared monoterpene compounds, such as α- and β-pinene, camphene, α-phellandrene, and terpinene, while the dominant compounds differed. D-camphor represented the main constituent in most of the works [5,7], whereas some authors reported 1,8-cineole or linalool as the main compounds [16,18]. These differences have been investigated in detail by Wanyang et al. [19], who defined the existence of five chemotypes for *C. camphora*, indicated as camphor, linalool, 1,8-cineole, borneol and (*E*)-nerolidol, based on the main essential oil compound.

Considering the current state of the art and the gaps in the scientific literature, we here performed a multi-scale survey on *C. camphora*, cultivated in Northern Italy investigating: (i) the micromorphology and the histochemistry of the secretory structures of young stems and leaves; (ii) the phytochemistry through the characterization of the essential oil (EO) profiles from different plant parts and following different conservation procedures; and (iii) the antibacterial activity of the leaf essential oils.

## 2. Results

### 2.1. Micro-Morphological Investigation

**Figure 1:**
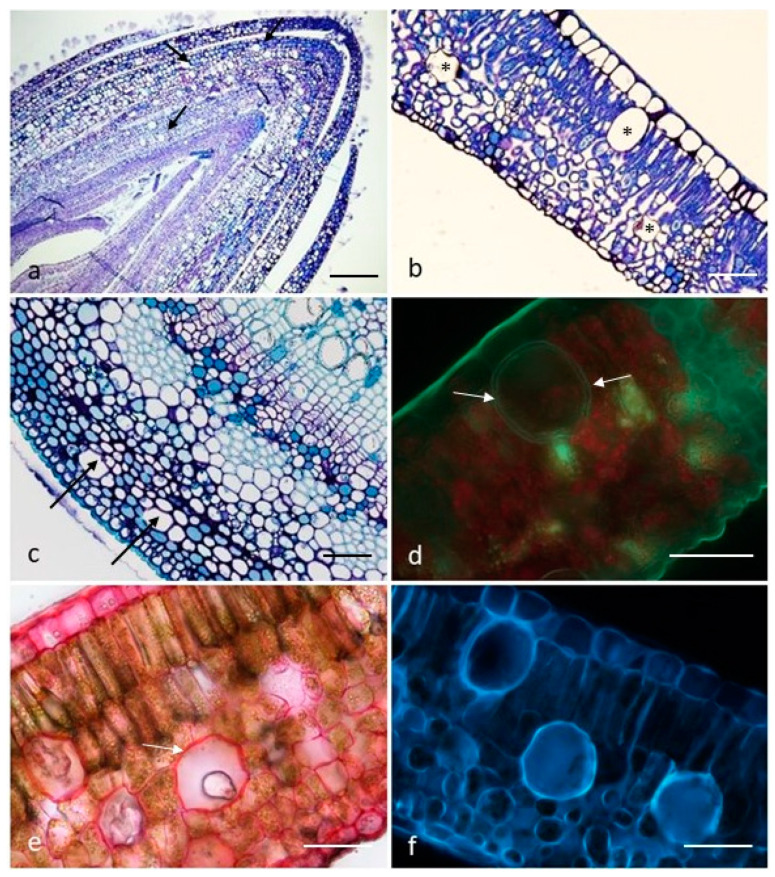
(**a**–**c**). Transverse sections of a leaf bud (**a**), a full-expanded leaf (**b**) and a young stem (**c**) showing the distribution pattern of the secretory cells (arrows and asterisks). Toluidine Blue. (**d**–**f**). Transverse sections of full-expanded leaves: primary fluorescence under UV light (**d**), notice the two-layered cell wall (arrows); Ruthenium Red test on the secretory cell walls (arrows) (**e**); primary fluorescence under Blue light (**f**). *Scale bars* = 200 µm (**a**); 100 µm (**c**); 50 µm (**b**,**d**–**f**).

Several secretory cells were observed in the leaves and young stems (Figure 1a–c). These cells were very abundant in leaf buds (Figure 1a), whereas the density was reduced in the full-expanded leaves (Figure 1b).

The palisade parenchyma cells were located immediately below the epidermis and were ellipsoidal in shape; the spongy mesophyll cells appeared globular in shape and were mostly distributed at the transitional region with the palisade parenchyma or adjacent to the abaxial epidermis (Figure 1b). In the young stems, these cells displayed a smaller diameter and a globular shape and occurred in the bark, in the cortical parenchyma, or were associated to the xylem medullary rays (Figure 1c).

The secretory cells were characterized by a multi-layered wall (Figure 1d) of variable thickness (4–6 μm if visible); it may appear continuous and uniform along the whole cell profile, as well as discontinuous or absent. The cell lumen appeared sporadically empty, and it was generally occupied by colorless, or pale yellow, heterogeneous secretory material.

The components of the cell wall layers were evidenced following the treatment with the PAS reaction, which highlighted total polysaccharides, and with the Ruthenium Red test for pectins (Figure 1e). Moreover, an intense primary fluorescence was observed as operating under UV and blue lights; this evidence indicated the presence of suberin in one of the wall layers (Figure 1f).

**Figure 2:**
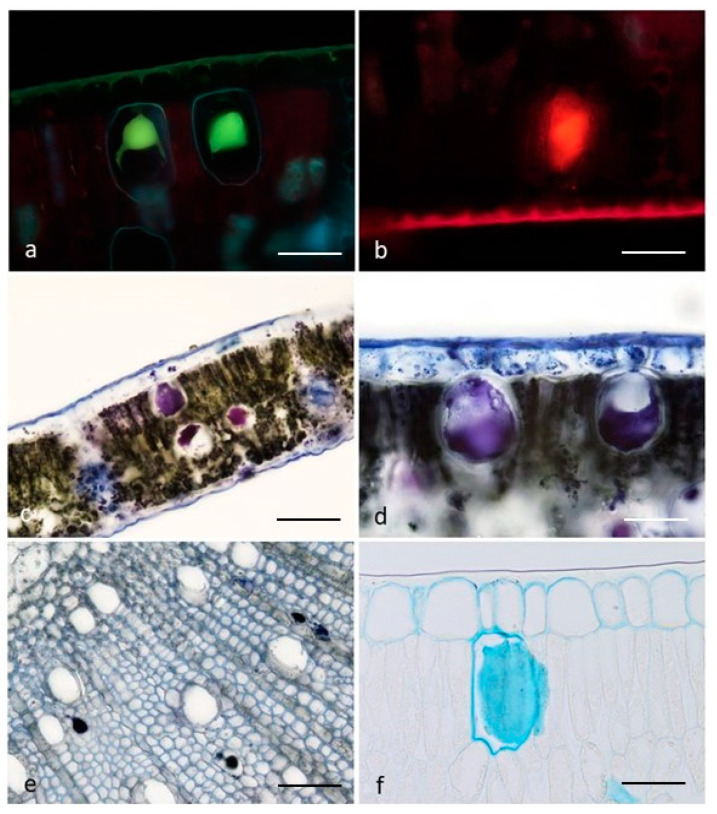
Results of the histochemical investigation on the secretory material of the oil cells (**a**–**e**) and of the mucillage cells (**f**): Fluoral Yellow-088 (**a**); Nile Red (**b**); Nadi reagent in leaves (**c**,**d**) and young stems (**e**); Alcian Blue (**f**). *Scale bars* = 50 µm (**a**,**b**,**d**–**f**); 100 µm (**c**).

Two types of secretory cells were distinguished: oil cells and mucilage cells (Table 1). In the oil cells, the content resulted in positive to the Fluoral Yellow-088 and Nile Red tests (Figure 2a,b; Table 1), evidencing the occurrence of abundant total lipids and neutral lipids, respectively. In particular, the application of the Nadi reagent produced an intense blue-violet coloration of the secretory material, both in the leaves and in the young stems (Figure 2c–e, Table 1), indicating the synthesis and storage of terpenoids. In the mucilage cells, the content resulted in positive exclusively to the Alcian Blue test, which is specific for mucopolysaccharides (Figure 2f; Table 1).

### 2.2. Phytochemical Investigation

The essential oil chemical profiles of *C. camphora* were evaluated over two successive years (2017 and 2018), and in 2018, according to different conservation procedures of the starting plant material, to assess the variability in their compositions (Table 2).

The 2017 essential oil, derived from the whole aerial parts (young stems, leaves and fruits), was obtained with a yield equal to 2.39%. Thirty-three different compounds were identified in total, with dominance of the monoterpene components, consisting mainly of oxygenated derivatives (63.0%), and to a minor extent, of hydrocarbons (29.0%); the minor fractions consisted of sesquiterpene hydrocarbons (1.2%), oxygenated sesquiterpenes (3.0%), and phenylpropanoids (1.9%) (Table 2). The most abundant compound was 1,8-cineole (*13*, 38.5%), followed by α-terpineol (*22*, 17.8%) and sabinene (*4*, 15.6%) (Table 2). To well understand the origin of these differences, in 2018, different plant aerial parts (leaves, young stems, bark, and fruits) were separately sampled and subjected to the drying process. Moreover, to evaluate whether the level of variability could depend on the preservation method, the leaves were subjected to different treatments before distillation: storage at −20 °C (*FL20*-EO), storage at −80 °C (*FL80*-EO), drying and storage at room temperature (*DL*), distillation as fresh material immediately after harvesting (*FL*).

Firstly, considering the essential oil profiles from leaves subjected to different conservation procedures (Table 2), a range of 16 (DL) to 19 (FL) total compounds were identified, with a prevalence of monoterpene hydrocarbons and oxygenated derivatives. The most abundant compound was 1,8-cineole (*13*, 46.6–63.6%), followed by sabinene (*4*, 19.7–23.3%) and *α*-terpineol *(22*, 1.1–13.6%). The comparison between essential oils from fresh leaves and those derived from samples stored at low temperatures showed quantitative variations for all the detected 19 compounds. The most significant, even if limited, variation was found for *α*-terpinene (*8*), with a relative abundance ranging from 0.4% in *FL*-EO up to 0.9% in both the low-temperature storage methods, for *γ*-terpinene (*16*) (0.9% in *FL20*-EO, 1.5% in *FL80*-EO) and terpinen-*4*-ol (*21*) (1.7% in *FL*-EO, 2.7% in *FL80*-EO). Fresh leaves essential oil finally showed an exclusive compound, *cis*-sabinene hydrate (*17*), present in very low amount (0.1%).

The comparison between the essential oils from dried and fresh leaves evidenced some qualitative variations among the minor compounds, with the absence of *cis*-sabinene hydrate *(17)*, *α*-terpinyl acetate (*27*) and *δ*-cadinene (*36*) in *DL*-EO. The increase of 1,8-cineole (*13*) was particularly significant, with the relative percentages moving from 46.6% in *FL*-EO up to 63.6% in *DL*-EO, and the concomitant decrease of *α*-terpineol (*22*), from 13.6% in *FL*-EO to only 1.1% in *DL*-EO. This variation suggests that during the drying process biosynthetic transformation pathways can be established, as already reported in the literature for other plant species [20]. This hypothesis was also supported by the evaluation of the relative amounts of both compounds in the essential oil profiles from leaves stored at −20 °C and −80 °C. In *FL80*-EO, they showed concentrations comparable to those of the fresh samples (Table 2), indicating that the transformation process was prevented. However, the storage at −20 °C seems not to be enough to prevent this process, since a slight increase in the amount of 1,8-cineole (*13*) was detected.

Therefore, we compared the essential oil profiles derived from different plant parts, showing significant qualitative and quantitative variations.

With regards to the young stems, already present in the sampling of 2017, the essential oil composition showed a qualitative overlapping with the *FL*-EO profile, with the dominance of 1,8-cineole (*13*) (53.7%), followed by terpinen-4-ol (*21*) (7.1%), sabinene (4) (6.7%) and *α*-terpineol (*22*) (5.7%). As for *DL*-EO, the high concentration of 1,8-cineole in the young stems was accompanied by a limited concentration of *α*-terpineol; the relative percentages of terpinen-4-ol (*21*), *γ*-terpinene (*16*) (4.0%), *α*-terpinene (*8*) (2.8%), *α*-phellandrene (*7*) (2.1%), terpinolene (*18*) (0.8%) and *p-*cymene (*9*) (1.5%) were instead higher than those in *FL*-80.

Concerning the bark essential oil, qualitative and quantitative differences emerged in comparison to the young stems profile. A relevant percentage of 1,8-cineole (*13*) (68.6%) was detected to be even higher than that of *DL*-EO. The other main compounds were terpinen-4-ol (*21*) (6.2%) and *α*-terpineol (*22*) (6.1%). The bark essential oil was also characterized by the occurrence of exclusive sesquiterpenes such as (*E*)-caryophyllene (*29*), *α*-humulene (*30*), spathulenol (*38*), humulene epoxide (*40*) and *epi*-*α*-cadinol (*41*), all occurring in relative amounts lower than or equal to 0.3%, except for *α*-humulene reaching 1.1%.

Finally, we examined the essential oil profile from fruits. Twenty-one different compounds were found in total, with 1,8-cineole (*13*) (40.2%), *α*-terpineol (*22*) (22.0%) and sabinene (*4*) (11.7%) as the major ones. All these compounds were already detected in the essential oils from all the other analyzed plant parts. Three exclusive compounds occurred in this sample: safrole (*25*) (5.1%), (*E*)-*β-*ocimene (*15*) (0.4%), and carvone (*24*) (0.1%).

### 2.3. Antibacterial Activity

The results of the antibacterial activity tests are reported in Table 3. The 2017 essential oil from the aerial parts of *C. camphora* showed a discrete inhibitory activity towards all the tested bacterial strains (MIC equal to 25 mg/mL, with the best MIC value of 12.5 mg/mL against one strain). The most promising results were found for *Listeria monocytogenes* with MIC values equal to 12.5 mg/mL. The bactericidal activity was equal to 50 mg/mL for all the strains, except for *Salmonella tiphymurium* with MBC values equal to 25 mg/mL. As a whole, the 2017 essential oil displayed a moderate inhibitory activity and a high antibacterial activity towards all the examined bacterial strains.

## 3. Discussion

The micro-morphological and histochemical investigations performed on leaves and young stems of *C. camphora* showed the presence of two types of secretory cells, oil cells and mucilage cells, with a distribution pattern consistent with the literature data. The investigation regarding the composition of the different layers of their wall allowed us to highlight the presence of a pectic-cellulosic layer and a suberin layer in both the cell types [21,22]. The histochemical analysis, in agreement with the results reported by Ravindran et al. [1] and Geng et al. [14], revealed that oil cells are the sites of production and accumulation of essential oils in *C. camphora.*

The characterization of essential oils obtained from diverse plant parts was then performed to evaluate differences among the profiles. The first screening was obtained with essential oil deriving from the complete plant aerial parts, including leaves, young stems and fruits, and showed the predominance of monoterpenes over all the other components. The most abundant compounds were 1,8-cineole (*13*), α-terpineol (*22*) and sabinene (*4*) (Table 2). The preliminary comparison with the literature data showed a moderate quantitative variability of the detected components, as well as a high heterogeneity due to the conservation and distillation procedures applied to the plant material by the various authors. Therefore, we evaluated, in more detail, the essential oil profiles obtained from individual plant parts.

The analyzed essential oils provided heterogeneous yields ranging from 0.09% for the bark up to 3.35% for the fruits, with leaf oil yields ranging between 0.62 and 1.40, proving that the value of the oil yield obtained in 2017 could depend on the fact that aerial parts were hydro-distilled as a whole.

The leaf essential oil profiles showed that the conventional conservation procedure by drying involved variations in the compositions due to the potential establishment of different metabolic processes; indeed, the drying procedures seemed to induce the transformation of α-terpineol into 1,8-cineole [20]; the same behavior was found in the samples stored at −20 °C. This evidence suggested that, after separation of the leaves from the plant, these processes might replace or prevail on the biosynthetic pathways occurring in the intact plant.

The comparison with literature data allowed us to point out some considerations. Most of the previous analyses on the leaf essential oil compositions revealed D-camphor as the main compound, with relative percentages ranging between 36.5% [6] and 73.8% [5], both in dried samples [6] and in samples subjected to unspecified treatments [6]. The work by Shujiang et al. [7], Chalchat and Valade [18] and Stubbs et al. [23] constituted exceptions, since the dominant compounds were represented by *endo*-borneol, a D-camphor precursor, in the former, and 1,8-cineole in the latter two, respectively. If we considered the essential oil composition reported by Chalchat and Valade [18], further qualitative and quantitative affinities emerged with the essential oil profile obtained herein from the dried leaves. The analysis of data reported in these two works showed that the relative amounts of most of the identified components had comparable concentrations. On this basis, although these authors did not report a detailed description of the preservation procedure of the plant material, it is reasonable to venture that the material was subjected to drying and stored at room temperature, following the most common practice. Therefore, we can assume that the species investigated herein belongs to the 1,8-cineole chemotype, since this compound dominated the profile, instead of D-camphor.

The work of Stubbs et al. [23], which discuss the comparison between essential oils obtained from entities belonging both to camphor and 1,8-cineole chemotypes, seems to validate this hypothesis, since the data we obtained for the fruit and leaf essential oils are in agreement with those reported in that paper. This work also shows that the oils obtained from the adult parts of the plant (trunk and adult branches) retain the presence of D-camphor, while the young parts seem to have none, suggesting that the absence of this compound in the essential oilswe obtained should be due to the age of plant parts we decided to analyze.

The comparison of the essential oilprofiles obtained from the other plant parts with the literature is less significant, due to the scarcity of contributions: Jiang et al. [17] investigated twigs and seeds; Shujiang et al. [7] studied branches and flowers, Stubbs et al. examined branches and trunk [23]. Furthermore, all these works reported D-camphor as the main or second main essential oilcompound, suggesting that the examined plants belong to different chemotypes with respect to our samples.

Regarding the essential oilexclusive compounds present in significant relative amounts in the analyzed plant parts, the bark is characterized by α-humulene which displays antitumoral [24], anti-inflammatory [25] and analgesic [26] effects, as well as the inhibiting capacity of CYP3A4, one of the main enzymatic complexes involved in the hepatic metabolism of drugs [27]. Safrole, exclusive of the essential oil from the immature fruits and present in high amounts, was already detected by Shanshan et al. [3] and Jiang et al. [18] and is currently recognized as a carcinogenic agent [28]. This compound is generically considered to be a typical component of the brown camphor oil from bark, not detected herein, and was widely used in the therapeutic field.

Concerning the ecological roles, α-humulene is produced by *Lantana camara* L. flowers to attract early-instar juveniles of the jumping spider *Evarcha culicivora* (Salticidae), which feed on nectar [29]. α-Humulene also exhibits acaricidal [30] properties. Safrole possesses antifungal properties, presumably to protect seeds; it occurs along with other compounds with xanthotoxin or additional toxic furanocoumarins in the plants of the families Apiaceae and Rutaceae. It was documented that these compounds have a phytosynergistic effect on the toxicity of xanthotoxin to *Heliothis zea* Boddie, a major agricultural pest [31].

For what concerns the biological activity of the three major common compounds across all the analyzed plant parts, 1,8-cineole, which accumulated in particular in the leaves after drying, plays a major role in the treatment of upper and lower airway diseases due to its anti-inflammatory properties, and appears to be active against antibiotic-susceptible and antibiotic-resistant pathogens [32,33,34]. In vitro experiments suggest anti-nociceptive properties [35] and a protective effect against ethanol-induced gastric mucosal damage and liver failure [36]. α-Terpineol, which prevailed in the fruits, shows antihypertensive and antiproliferative effect on human erythroleukemic cells [37,38], as well as antioxidant, anticancer, anticonvulsant, antiulcer, anti-nociceptive, and anti-inflammatory actions [39,40,41,42,43]. Finally, sabinene, principally abundant in the leaves, is known to exhibit anti-inflammatory properties [44]. In particular, Matias et al. reported that sabinene extracted from *Cordia verbenacea* leaves was able to enhance the effect of aminoglycosides [45].

Regarding the ecological role, 1,8-cineole is recognized as an attractant of different bees and is also an alarm pheromone for *Bombus terrestris* L. [46,47,48]. In particular, it is considered a floral attractant of the male euglossine bees (tribe Euglossini, family Apidae) in many genera of orchids distributed in Central and Southern America [49]. It is also applied as an insecticide and insect repellent [50] and possesses a direct anti-bacterial activity [51]. α-Terpineol exhibits insecticidal properties and its emission from the bark of *Pinus sylvestris* L. was documented to deter the black pine sawyer beetle *Monochamus galloprovincialis* Olivier, a serious pest of the tree [52]. Sabinene possesses anti-fungal activities against some pathogens of *Citrus* spp. [53]. It is produced by fungi of the genus *Phomopsis*, residing endophytically in *Odontoglossum* spp., and might be triggered to activate a defense mechanism of the host plant for self-protection [54].

Moreover, considering the antibacterial potential of essential oilss, a previous study on *C. camphora* reported that the leaf essential oilfrom China is a good antibacterial agent, exhibiting a wide range of antibacterial activities against common bacteria, e.g., *Chromobacterium violaceum*, *Staphylococcus aureus*, *Escherichia coli* and *Pseudomonas aeruginosa* [55]. These authors also investigated the anti-quorum sensing (anti-QS) inhibitory activity of the EO. It significantly decreased the production of violacein and biofilm biomass in *C. violaceum*, and inhibited the biofilm formation and swarming movement, independent of affecting the bacterial growth. Moreover, several contributions from the literature [56,57,58] report that the antibacterial activity of essential oils from different sources is primarily provided by the family of oxygenated monoterpenes, and in particular by 1,8-cineole. Due to its relative high abundance in essential oilss from *C. camphora* investigated, the antibacterial action we found could be attributed to the preponderant presence of this compound.

Collectively, the prominent antibacterial activity and anti-QS activities clearly support that *C. camphora* EO acts as a potential antibacterial agent and QS inhibitor in the prevention of bacterial contamination. Hence, *C. camphora* essential oil may represent an alternative source of natural antimicrobial substances for use in food systems to prevent the growth of food-borne bacteria, thus extending the shelf-life of the processed food. The antibacterial action of the leaf oils was also documented against *Pasturella multocida*, cause of a range of diseases in mammals and birds, and against the respiratory pathogen *Haemophilus influenza* [59].

## 4. Materials and Methods

### 4.1. Plant Material

The sampling for the micro-morphological, phytochemical and biological activity analyses was performed in late spring 2017 and early summer 2018 on a unique entity conserved at the Ghirardi Botanic Garden in Toscolano Maderno (Brescia, Italy). The amount of material collected was assessed on site, depending on the state of the plant and in accordance with the phase of its phenological cycle. Voucher specimens, labelled with the codes GBG2017/037 and GBG2018/056 and GBG2018/057, were stored in the Herbarium of the Ghirardi Botanic Garden.

### 4.2. Chemicals

Solvents and reagents were purchased from Sigma Aldrich (Merck group, Milan, Italy) and used as received.

### 4.3. Morphological Investigation

#### Light Microscopy (LM)

The plant samples, embebbed and fixed in historesin, were sectioned and treated with the following dyes: Fluoral Yellow 088 and Sudan III-IV for total lipids [60], Nile Red for neutral lipids [61], Nadi reagent for terpenes [62], PAS reaction for total polysaccharides, Ruthenium Red for pectins [63], Alcian Blue for mucopolysaccharides [64] and Naturstoff Reagenz-A for flavonoids [64]. Control procedures were performed concurrently for all the employed histochemical staining. Primary fluorescence was also evaluated under UV and blue lights.

Observations were carried out under a Leitz DM-RB Fluo optic microscope.

### 4.4. Phytochemical Investigation

#### 4.4.1. Essential oilIsolation

In 2017, the whole aerial parts (young stems, leaves and fruits) were collected and air-dried. The samples (300 g) were reduced into small pieces, then immersed into 10 L flasks filled with 6 L of deionized water and subjected to hydrodistillation using a Clevenger-type apparatus for 4 h.

In 2018, the different plant parts (leaves, young stems, bark and immature fruits) were separately sampled and air-dried. The leaves were also subjected to different preservation procedures: storage at −20 °C, storage at −80 °C, air-drying and storage at room temperature, distillation as fresh material immediately after harvesting. All the conservation procedures were applied for a period of three months from the collection date.

The fresh and air-dried samples of *C. camphora* of the year 2018 were reduced into small pieces, then immersed into 4 L or 2 L flasks filled with deionized water and subjected to hydrodistillation using a Clevenger-type apparatus for 2 h. The solvent-to-sample ratio used for all the Clevenger distillations performed was of 1 L of water for 50 g of sample.

Once obtained, the essential oilwas decanted and separated from water, which residual drops were removed using anhydrous sodium sulphate. The oil yield was estimated on a fresh weight basis (*w*/*w*) for fresh samples and on a dry weight basis (*w*/*w*) for the dried ones.

#### 4.4.2. GC-MS Analysis

Essential oilswere analyzed by GC-MS using an Agilent 6890 N (Agilent Technologies SpA, Cernusco s/N, Milan, Italy) equipped with a 5973 N mass spectrometer. Separation was achieved on an HP-5 MS capillary column (5% phenylmethylpolysiloxane, 30 m, 0.25 mm i.d., 0.1 μm film thickness; J & W Scientific, Folsom, CA, USA) using helium as the carrier gas (1 mL min^−1^). The temperature of the oven was set to at 60 °C for 5 min, then raised at 4 °C min^−1^ up to 220 °C, finally 11 °C min^−1^ up to 280 °C. The TICs were acquired at 70 eV scanning in the 29–400 *m*/*z* range. The oil samples were diluted 1:100 in *n*-hexane, and the volume injected was 2 μL (three replicates). Data were analysed using MSD ChemStation software (Agilent, Version G1701DA D.01.00) and the NIST Mass Spectral Search Program for the NIST/EPA/NIH Mass Spectral Library v. 2.0. The identification of essential oil components was performed by a comparison of retention indices, calculated using a C7–C30 series of *n*-alkanes (Merck, Milan, Italy) and mass spectra of unknown peaks with those contained in the commercial libraries WILEY275, NIST 17, ADAMS and FFNSC3, as well as those in a homemade library. Percentage values of essential oil components were obtained from the peak areas in the chromatogram without the use of correction factors.

### 4.5. Antibacterial Activity

#### 4.5.1. Bacterial Strains Employed

The following strains belonging to American Type Culture Collection (ATCC; LGC Standards S.r.l., Sesto San Giovanni, Milan, Italy) have been taken into account for the determination of antibacterial activity: *Staphylococcus aureus* ATCC 6538; *Enterococcus faecalis* VAN B V583 E; *Listeria monocytogenes* ATCC 7644; *Escherichia coli* ATCC 15325; *Pseudomonas aeruginosa* ATCC 27853; *Salmonella tiphymurium* ATCC 14028.

Bacterial strains stored at −80 °C in glycerol suspension were sowed on plates containing tryptic soy agar (TSA—Thermo Fisher Scientific, Milan, Italy) and incubated overnight at 37 °C. Subsequently, one colony from these cultures was inoculated in a culture broth of brain heart infusion and incubated at 37 °C for 24 h to obtain freshly cultured microbial suspensions.

#### 4.5.2. Minimum Inhibitory Concentration (MIC) and Minimum Bactericidal Concentration (MBC) Determinations

Twofold serial microdilution method was used for the determination of the Minimum Inhibitory Concentration (MIC) values of essential oil against each strain. Essential oil was diluted with BHI (Brain Hearth Infusion—Thermo Fisher Scientific, Milan, Italy) broth supplemented with dimethyl sulfoxide (DMSO) as a solvent to a final ratio of 1:3:4 (EO:DMSO:BHI, *v*/*v*). The tested dilutions ranged from 100.00 mg/mL to 1.5625 mg/mL of EO. Determination of MIC was performed in a final volume of 200 μL in 96-well polypropylene microtiter plates according to Fratini et al. [65].

Microplates were incubated at 37 °C ± 2 °C for 24 h in a humid chamber. The MIC value was defined as the lowest concentration of essential oilneeded to visibly inhibit bacterial growth [66]. The Minimum Bactericidal Concentration (MBC) was determined by plating on Tryptone Soy Agar (TSA) from a microplate well corresponding to a MIC value to the higher concentration tested. TSA plates were incubated at 37 °C ± 2 °C for 24 h. MBC value was defined as the lowest concentration of essential oilthat corresponds to the sample in which no bacterial growth was visible. MIC and MBC determinations were performed in triplicate and the results were expressed as mg/mL of EO. Mode of the triplicate was employed for value comparisons.

## 5. Conclusions

The investigation presented in this work and conducted on three different areas of analysis has primarily allowed us to describe the micromorphological characteristics of plant parts analyzed. The comparative study conducted by light microscope images, on leaf buds, fully expanded leaves and young stems and correlated to a novel in-depth histochemical investigation has revealed a pattern of distribution of secretory cells, which can be divided into oil cells and mucilage cells, confirming previous literature data.

However, the phytochemical investigation of the composition of the essential oilss obtained in the following two years (2017 and 2018) and from different plant matrices (leaves, young stems, bark, immature fruits) highlighted the probable belonging of the studied entity to the 1,8-cineole chemotype. Comparison of all the essential oils obtained here from *C. camphora* showed the peculiar absence of D-camphor, which has no comparison in the literature. It should therefore be interesting to study the production of volatile compounds from different parts of the plant depending on their aging.

Furthermore, this is the first report on the comparison of leaf essential oilsprofiles obtained following different preservation techniques (fresh, stored at −20 °C, stored at −80 °C, dried), and specifically, no previous work referred to the storage procedure at −80 °C. The obtained essential oilprofiles were compared with each other and with literature data, obtaining a high level of chemical variability; this allowed us to define the optimal conservation technique in relation to the overall yield of essential oil, i.e., storage at −20 °C, and to characterize the common and exclusive compounds. Regarding the desired quality of essential oil, the importance of conservation procedures emerged. In fact, in the case of an essential oil rich in 1,8-cineole it is advantageous to use the drying technique, while in the case of an essential oil rich in α-terpineol, it is preferable to start with fresh plant material subjected to freezing.

Finally, both the literature investigation conducted on the ecological and biological properties of essential oilsand the preliminary tests for the antibacterial activity of EO 2017 evidenced the potential insecticidal, antifungal and antimicrobial application of essential oilss towards various organisms dangerous for plant or human health, which should be the subject of further investigation.

## Figures and Tables

**Table 1:** Results of the histochemical analysis on the leaves and young stems of *Cinnamomum camphora*.

Test	Target Compound Class	Oil Cells		Mucilage Cells	
		Leaf	Stem	Leaf	Stem
Fluoral Yellow-088	Total lipids	++	++	−	−
Nile Red	Neutral lipids	++	++	−	−
NADI reagent	Terpenes	++	++	−	−
Alcian Blue	Acid mucopolysaccharides	−	−	++	++
Naturstoff Reagentz-A	Flavonoids	−	−	−	−

Symbols: (−) negative response; (++) intensely positive response.

**Table 2:** Composition of the essential oils of *C. camphora* obtained in 2017 and 2018 from different plant parts and conservation procedures. *FL*, fresh leaves; *FL20*, storage at −20 °C; *FL80*, storage at −80 °C; *DL*, dried leaves. Data are reported as relative abundance %.

*Compounds Description*				2017	2018						
*n*.	LRI ^a^	Name	Class	Aerial Parts	FL	FL20	FL80	DL	Young Stems	Bark	Fruits
*1*	921	α-thujene	HM	0.7	0.6	0.8	0.8	0.6	0.7	0.5	0.5
*2*	927	α-pinene	HM	4.3	5.0	5.4	5.4	3.7	5.0	2.6	3.1
*3*	940	camphene	HM	0.1	0.1	0.1	0.1	0.1	0.1	0.1	-
*4*	966	sabinene	HM	15.6	21.0	20.0	19.7	23.3	6.7	2.8	11.7
*5*	969	β-pinene	HM	3.7	4.5	4.8	4.7	4.2	4.9	2.6	2.9
*6*	990	myrcene	HM	1.1	1.1	1.2	1.1	0.8	0.9	0.4	0.9
*7*	1004	α-phellandrene	HM	0.8	0.7	0.8	0.9	0.5	2.1	-	2.6
*8*	1014	α-terpinene	HM	0.8	0.4	0.9	0.9	0.3	2.8	-	1.0
*9*	1022	*p*-cymene	HM	-	0.4	0.4	0.4	0.3	1.5	5.6	0.3
*10*	1023	*o*-cymene	PP	0.4	-	-	-	-	-	-	-
*12*	1025	sylvestrene	HM	0.8	-	-	-	-	-	-	-
*11*	1026	limonene	HM	-	1.3	1.3	1.3	0.8	2.3	1.3	1.6
*13*	1027	1,8-cineole	OM	38.5	46.6	48.1	46.4	63.6	53.7	68.6	40.2
*14*	1038	(*Z*)-β -ocimene	HM	-	-	-	-	-	-	-	0.1
*15*	1048	(*E*)-β -ocimene	HM	-	-	-	-	-	0.1	-	0.4
*16*	1056	γ-terpinene	HM	1.3	0.9	1.5	1.5	0.4	4.0	-	1.6
*17*	1065	*cis*-sabinene hydrate	HM	0.6	0.1	-	-	-	-	-	-
*18*	1085	terpinolene	HM	0.3	0.2	0.3	0.3	0.2	0.8	-	0.4
*19*	1118	*cis-p*-menth-2-en-1-ol	OM	0.1	-	-	-	-	-	-	-
*20*	1165	δ-terpineol	OM	0.5	0.3	0.2	0.3	0.1	0.2	0.1	0.3
*21*	1174	terpinen-4-ol	OM	2.7	1.7	2.5	2.7	0.1	7.1	6.2	4.2
*22*	1187	α-terpineol	OM	17.8	13.6	10.7	12.3	1.1	5.7	6.1	22.0
*23*	1192	*cis*-piperitol	OM	0.1	-	-	-	-	-	-	-
*24*	1239	carvone	OM	0.6	-	-	-	-	-	-	0.1
*25*	1283	safrole	PP	1.4	-	-	--	-	-	-	5.1
*26*	1343	α-cubebene	HS	-	-	-	-	-	-	0.1	-
*27*	1346	α-terpinyl acetate	OM	2.3	1.2	0.8	1.1	-	0.9	0.4	0.9
*28*	1405	methyl-eugenol	PP	0.2	-	-	-	-	-	-	-
*29*	1407	(*E*)-caryophyllene	HS	-	-	-	-	-	-	0.1	-
*30*	1440	α-humulene	HS	-	-	-	-	-	-	1.1	-
*31*	1475	α-selinene	HS	0.2	-	-	-	-	-	-	-
*32*	1486	bicyclogermacrene	HS	0.2	-	-	-	-	-	-	-
*33*	1492	α-muurolene	HS	0.1	-	-	-	-	-	-	-
*34*	1504	γ-cadinene	HS	0.1	-	-	-	-	-	-	-
*35*	1510	*trans*-calamenene	HS	-	-	-	-	-	-	0.1	-
*36*	1511	δ-cadinene	HS	0.6	0.3	0.1	0.2	-	0.2	0.1	0.2
*37*	1562	8-acetoxy-carvotan acetone	OS	1.5	-	-	-	-	-	-	-
*38*	1563	spathulenol	OS	-	-	-	-	-	-	0.3	-
*39*	1565	germacrene D-4-ol	OS	0.4	-	-	-	-	-		-
*40*	1593	α-humulene epoxide II	OS	-	-	-	-	-	-	0.3	-
*41*	1630	epi-α-cadinol	OS	-	-	-	-	-	-	0.2	-
*42*	1632	α-muurolol	OS	0.4	-	-	-	-	-	-	-
*43*	1638	β-eudesmol	OS	0.2	-	-	-	-	-	-	-
*44*	1642	α-cadinol	OS	0.6	-	-	-	-	-	0.2	0.1
		**EO yield (%)**		**2.39**	**1.17**	**1.38**	**1.40**	**0.62**	**0.37**	**0.09**	**3.35**
		**Monoterpenes (HM)**		**29.5**	**36.2**	**37.5**	**37.1**	**35.1**	**32.1**	**15.9**	**26.9**
		**Oxygenated monoterpenes (OM)**		**63.4**	**63.4**	**62.4**	**62.7**	**64.9**	**67.6**	**81.5**	**67.7**
		**Sesquiterpenes (HS)**		**1.2**	**0.3**	**0.1**	**0.2**	**-**	**0.2**	**1.5**	**0.2**
		**Oxygenated sesquiterpenes (OS)**		**3.0**	**-**	**-**	**-**	**-**	**-**	**0.1**	**0.1**
		**Phenyl propanoids (PP)**		**1.9**	**-**	**-**	**-**	**-**	**-**	**-**	**5.1**
		**Total**		**99.0**	**99.9**	**100.0**	**100.0**	**100.0**	**100.0**	**99.8**	**100.0**

The percentage values are means of two determinations (two preparations of essential oil dilution in *n*-hexane), with relative standard deviation values (RSD%) below 20% for all components. ^a^ Linear retention index (LRI), experimentally obtained on a HP-5MS column using a C_7_-C_30_ mixture of *n*-alkanes. ^b^ Other abbreviations: n. = number assigned to compound, HM = Monoterpene Hydrocarbons; OM = oxygenated monoterpenes; HS = sesquiterpene Hydrocarbons; OS = Oxygenated Sesquiterpenes; PP = Phenyl Propanoids.

**Table 3:** Results of the antibacterial activity tests on the 2017 EO of *Cinnamomum camphora*.

Activity	*Staphylococcus aureus*	*Listeria monocytogenes*	*Enterococcus faecalis*	*Salmonella tiphymurium*	*Escherichia coli*	*Pseudomonas aeruginosa*
**MIC values**	25.000	12.500	25.000	25.000	25.000	25.000
**MBC values**	50.000	50.000	50.000	25.000	50.000	50.000

MIC = Minimum Inhibitory Concentration; MBC = Minimum Bactericidal Concentration, both expressed in mg/mL.

## Data Availability

Not applicable.
